# The association of intimate Partner violence and decision making power on nutritional status of married women in Ethiopia: a multilevel mixed-effect analysis

**DOI:** 10.1186/s12905-023-02459-0

**Published:** 2023-06-13

**Authors:** Fentanesh Nibret Tiruneh, Peter Austin Morton Ntenda, Tinebeb Tamir

**Affiliations:** 1 Department of Applied Human Nutrition, Faculty of Chemical and Food Engineering, Bahir Dar Institute of Technology, Bahir Dar University, Bahir Dar, Ethiopia; 2 Malaria Alert Centre, Kamuzu University of Health Sciences, Private Bag 360, Blantyre 3, Chichiri, Malawi; 3 WaterAid Ethiopia, Addis Ababa, Ethiopia

**Keywords:** Intimate partner violence, Nutritional status, Women, Decision making, Community-level, Individual-level

## Abstract

**Background:**

A growing body of research has established a link between intimate partner violence (IPV) and decision-making autonomy on women’s mental, physical, and reproductive health consequences, as well as child nutritional status. However, there is a scarcity of research on the effects of IPV and decision-making autonomy on women’s nutritional status. To date, no research has been conducted in Ethiopia to investigate the impact of IPV and decision-making autonomy on women’s nutritional status. Therefore, the purpose of this study was to investigate the relationship between IPV and decision-making power at both the individual and community levels on women’s nutritional status.

**Methods:**

We analyzed data from the 2016 Ethiopian demographic and health survey. Our study focused on 3,660 married non-pregnant women of reproductive age. We used the chi-squared test and spearman correlation coefficients for bivariate analysis. The relationship between IPV and decision making power with nutritional status was evaluated using multilevel binary logistic regression models while controlling other determinants.

**Results:**

Around 28% of women reported at least one of the four types of IPV. Approximately 32% of women had no decision-making power at home. About 27.1% of women were underweight (BMI < 18.5), while 10.6% were overweight/obese (BMI ≥ 25). Women who had sexual IPV had a higher chance of being underweight (AOR = 2.97; 95% CI: 2.02–4.38) than women who had no sexual IPV. While women who had decision-making power at home were at lower odds of being underweight (AOR = 0.83; 95% CI: 0.69–0.98) than their counterparts. The findings also revealed a negative relationship between being overweight/obese and community-level women’s decision-making autonomy (AOR = 0.75; 95% CI 0.34–0.89).

**Conclusions:**

Our findings show the presence of a significant association between IPV & decision-making autonomy with women’s nutritional status. Therefore, effective policies and programs to end violence against women and encourage women to participate in decision-making are required. This will help to improve the nutritional status of women as well as the nutritional outcomes of their families. This study suggests that efforts to achieve Sustainable Development Goal (SDG5) may have an impact on other SDGs particularly SDG2.

## Background

Malnutrition encompasses both under-nutrition and over-nutrition, both of which can result in the development of diseases and chronic health conditions [1]. Women’s malnutrition during their reproductive years has a negative impact on maternal, neonatal, and child health outcomes [2]. For decades, high rates of underweight among women have been a major public health concern in low and middle income countries (LMIC). However, rising urbanization and globalization in LMICs, along with associated lifestyle and behavioral changes, have resulted in a significant shift in the epidemiological trend from underweight to overweight [3, 4]. Despite the fact that the prevalence of underweight remains high in Ethiopia, the trend of overweight/obesity in urban areas has increased dramatically from 10.9 to 2000 to 21.4 in 2016 [5, 6]. The nutritional status of women of reproductive age has a crucial influence on the health of women and the health of the next generation.

Previous research findings indicated that the major factors of nutritional status among women are thought to be linked to women’s socio-demographic, economic and reproductive health characteristics [7–10]. Nonetheless, the role of social and psychological factors in women’s nutritional status is understated. Intimate partner violence (IPV), which includes physical, emotional, and sexual abuse perpetrated against women by male intimate partners, is recognized as one of the major cultural, social, and mental threats that may influence women’s nutritional status [11]. The decision-making autonomy of women may also an important component of women’s nutritional status. Low women’s autonomy can have a negative nutritional effect on them [12]. Women’s low decision-making autonomy limits their ability to make decisions about the amounts and types of food fed to their children and themselves [13].

Gender equality, empowering women and girls, and improving nutritional status are essential components of the Sustainable Development Goals (SDGs), particularly SDGs 5 and 2, which may have an impact on other SDGs. Gender equality is intertwined with all 17 SDGs. The 2030 Agenda for Sustainable Development will not be completely realized until gender inequality is addressed [14].

IPV and women’s decision-making autonomy are linked to women’s nutritional status via community norms and practice [15]. According to research, the decision-making abilities of women in a community may have an impact on their health and health-related behavior [16]. A growing body of research has established a link between IPV and decision-making autonomy, with various aspects of women’s mental, physical, and reproductive health consequences, as well as child nutritional status [17–20]. However, there is a scarcity of research on the effects of IPV and decision-making autonomy on women’s nutritional status. Furthermore, these previous studies only looked at individual-level factors. To date, no research in Ethiopia has been conducted to investigate the relationship between IPV and decision-making autonomy on women’s nutritional status. The purpose of this study was to investigate the relationship between IPV and decision-making power at both the individual and community levels on women’s nutritional status in a nationally representative sample of Ethiopia, where malnutrition is prevalent. This study provides information for interested stakeholders and policymakers to understand the relationship between IPV and women’s decision making on their nutritional status, which may help to achieve the SDGs particularly SDG 5 and 2 concurrently.

## Materials and methods

### Data sources

The data for this study came from the 2016 Ethiopia Demographic Health Survey (EDHS), conducted by the National Central Statistical Agency (CSA) at the request of the Federal Ministry of Health from January 18, 2016, to June 27, 2016. A stratified multistage cluster sample of 645 clusters was constructed (202 in urban areas and 443 in rural areas). The primary sampling units were derived from the sampling frame created for the 2007 Ethiopian Population and Housing Census. The 2016 EDHS defined a cluster as a census enumeration area covering 181 households on average. Face-to-face structured interviews were conducted. Before the survey, a pretest was done to ensure that the questions were clear for the participants [21].

**Fig. 1:**
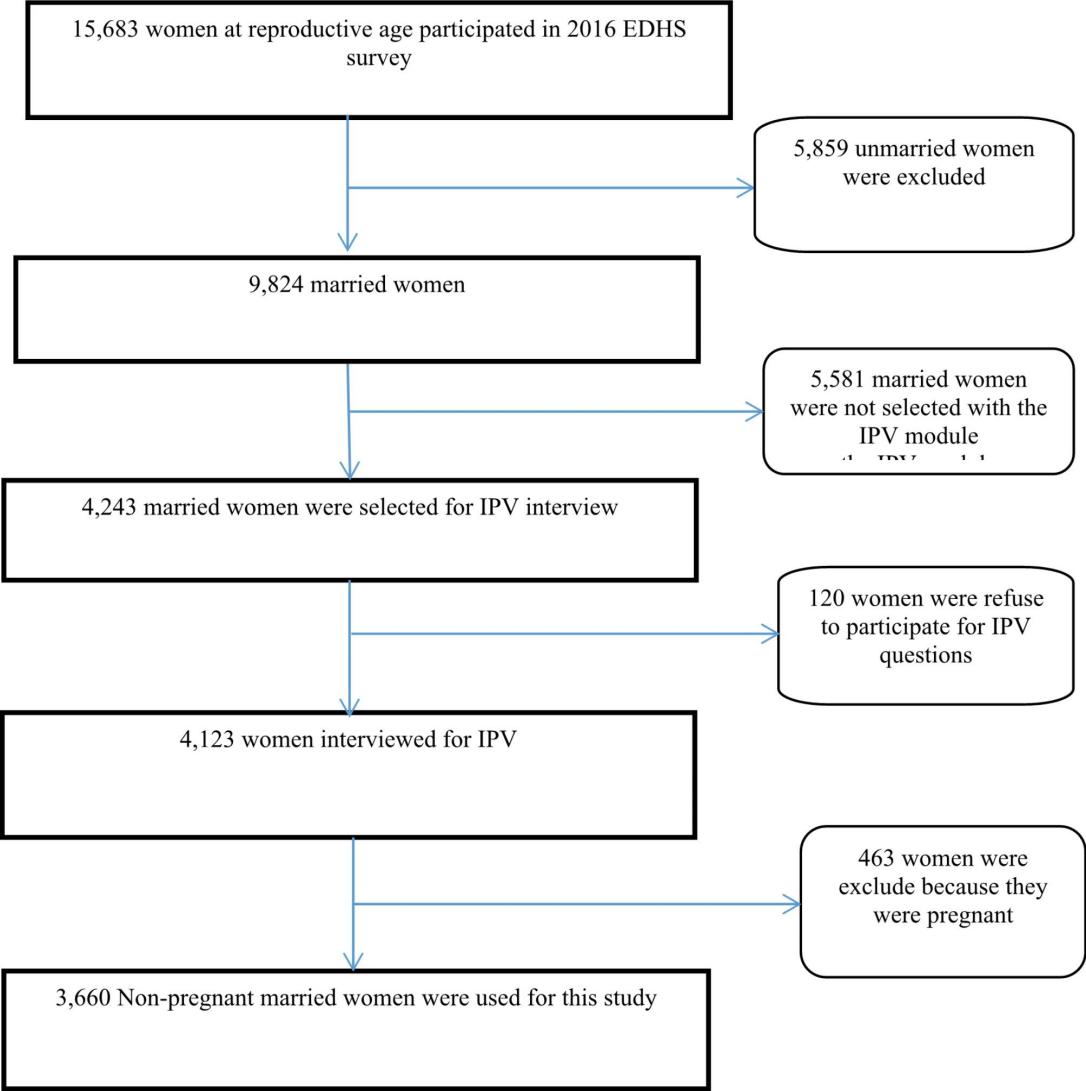
Sampling procedure of study participants

Of the 15,683 women aged 15–49 years 9,824 were married. One woman from each household was selected at random for the domestic violence module in the women’s questionnaire to answer an additional set of questions regarding IPV perpetrated by her husband. Among 4,243women eligible to respond to the domestic violence module, 120 women refused to participate in the IPV questions. An additional 463 pregnant women were excluded because anthropometric measurements like body weight, which increases rapidly during the pregnancy, may reduce the specificity of the indicator. Our analyses focused on 3,660 non-pregnant married women of reproductive age (15–49 years) (Fig. 1).

### Outcome measure

The 2016 EDHS measured the height and weight of all women aged 15–49 years. Trained investigators weighed each woman by using a lightweight seca. The body mass index (BMI), calculated as weight in kilograms divided by height squared in meters, was used to assess the nutritional status in this study. We chose BMI to assess under nutrition and overweight/obesity of women because it does not require a reference table from a well-nourished population. A cutoff point in the BMI of 18.5 kg/m^2^ was used to define under nutrition and BMI 25 kg/m^2^ or above is used to define overweight/obese as recommended by World Health Organization [22].

### Individual-level explanatory variables

The two groups of explanatory variables in our study were women’s experience of IPV and decision making power.

The 2016 EDHS used a standardized approach to the measurement of IPV. This approach involves implementing the most valid measures, shortened and modified version of the Conflict Tactics Scale (CTS-2). The modified Conflict Tactics Scales (CTS2) is the most widely used instrument for measuring intimate partner violence. The CTS-2 approach of inquiring about a wide range of acts has the additional advantage of giving the respondent multiple opportunities to disclose any experience of IPV and thus increases the validity of measurement [23].

In our study, IPV was measured using twelve “yes” or “no” questions that asked women if they had ever experienced one or more violent occurrences at the hands of their husband or partner in their lifetime. [23–25].

In addition to IPV, we included women’s decision-making autonomy as the key explanatory variable because it is closely related to women’s health and health-related issues. Evidence suggests that women’s ability to engage in decision-making over some significant things (such as big household purchases or personal health care) is essential for practicing good health-seeking behaviors, including healthy nutritional status [26].

Decision-making power was measured by asking the respondents whether they were involved in finalizing decisions in the following four situations: personal health care (personal decision making power), large household purchases (economic decision making power), visits to family or relatives (mobility decision making power), and how to spend money (economic decision making power). Because few women in our sample made decisions individually, we constructed a binary variable (0 = only husband/partner made decisions and 1 = respondent alone or joint decision between respondent and husband/partner), indicating at least partial involvement on the part of women in the decision-making process.

### Community-level explanatory variables

Based on the aggregation of individual responses for each item at the community (cluster) level, we assessed community-level explanatory variables for women’s nutritional status which are the proportions of women who had experienced any type of IPV in the community and the proportion of women who had women’s decision making power in the community. These community-level variables represented community norms and social circumstances concerning women’s autonomy, violence against women, and women’s health status, including nutritional status [27].

### Control variables

Based the existing literatures, we included numerous socioeconomic and demographic variables in our analyses because they are critical to understanding the relationship between IPV and women’s decision making power on their nutritional status [28, 29]. We classified participants by their current age: younger (15–24 years), middle aged (25–34 years) or older (35–49 years). The place of residence was categorized as urban versus rural. The women’s educational level was defined as: no education, primary, secondary or higher. Religion was categorized as Orthodox, Muslim, protestant and others. Employment status was categorized as no versus ye), Total number of children was measured as 0, 1–2, 3–4 and 5. Having ever used contraception was categorized as yes vs. no. women’s wealth index was categorized into five quintiles: poorest, poor, middle, rich, and richest. In addition we included community-level female education which was categorized as to whether a high or low proportion of females had a secondary or higher educational level in the community.

### Statistical analyses

All analyses were performed using Statistical Analysis Software (SAS) (version 9.4). We conducted a series of bivariate analyses by using chi-squared tests to examine the association of explanatory with outcome variables. We also calculated Spearman correlation coefficients to analyze the associations among the community-level characteristics. We then used SAS’s GLIMMIX command to perform multilevel binary logistic regression analyses to examine the relationships between IPV and women’s decision-making power on women’s nutritional status while controlling for covariates.

### Model building approach

Four models were developed. Model1 (empty model) included the outcome variable only to examine the variation in nutritional status among communities. Model (model with only individual-level variables), Model three (model with only community-level variables) and the final multilevel model (model 4) with both individual level and community level variables fitted at the same time with nutritional status. The fixed effects for the multilevel binary logistic regression model were reported as adjusted odds ratios (AORs) with 95% confidence intervals (CI). The intraclass correlation coefficient (ICC) was used to analyse the random effects. ICC represents the variation in women’s nutritional status among communities. The Akaike information criterion (AIC) was used to measure the goodness of fit of each model; a lower number implies that the model is better fit.

### Ethical issues

This manuscript only involved secondary analyses of available the EDHS data. The EDHS data collection methods were carried out in accordance with relevant guidelines and regulations. The survey received ethical approval from the Ministry of Innovation and Technology the Federal Democratic Republic of Ethiopia, and the International Classification of Functioning Institutional Review Board. Before the interviews, all participants provided written informed consent. A parent or guardian gave permission for their adolescent to participate in the study. The authors obtained permission from the DHS program to use the data: https://dhsprogram.com/data/dataset/Ethiopia_Standard-DHS_2016.cfm.

## Results

About 28.4, 18.6, 20.7, and 6.8% of married non-pregnant women in our sample had experienced any, physical, emotional, or sexual IPV, respectively. Approximately 32% of participants reported having no decision-making power at home. Most of the respondents (75.8%) resided in rural areas and were unemployed (65.8%). In terms of nutrition status, over 27% of women were underweight, 67.3% were normal weight, and 10.5% were overweight/obese (Table 1).

**Table 1:** Sample Characteristics (n = 3,660)

Characteristics		Percentage (%)
Intimate partner violence (IPV)		
	Physical IPV	18.6
	Emotional IPV	20.7
	Sexual IPV	6.8
	Any IPV	28.4
Decision-making autonomy		
	No	32.0
	Yes	62.9
Age group of the respondent (years)		
	15–24	23.5
	25–34	43.3
	35–49	33.1
Religion		
	Orthodox	37.1
	Muslim	42.3
	Protestant	18.8
	others	1.8
Place of residence		
	Urban	24.1
	Rural	75.8
Education level of respondent		
	No education	58.7
	Primary	27.6
	Secondary or higher	13.7
Employment status of respondent		
	Unemployed	65.9
	Employed	34.1
Total number of children ever born		
	0	8.3
	1–2	30.2
	3–4	25.4
	≥ 5	36.1
Contraceptive use		
	No	64.8
	Yes	35.2
Wealth index		
	Poorer	29.8
	Poor	16.1
	Medium	14.4
	Rich	13.0
	Richer	26.7
Nutritional status (BMI)		
	Underweight	27.1
	Normal	62.3
	Overweight/obese	10.5

Table 2 shows that an average of 27.1% of respondents in all communities had experienced any type of IPV (physical, sexual, or emotional). Approximately 64 and 28% of respondents had decision-making autonomy at home and had completed secondary or higher education, respectively. Overall, there was a statistically significant negative correlation between community-level IPV and decision-making autonomy (r = -0.15*).

**Table 2:** Pearson Correlation matrix among community-level characteristics (n = 636)

Characteristics	Mean ± SD	A	B	C
A. Percentage of women who had experienced any IPV ^a^	27.1 ± 24.8	1.0		
B. Percentage of women who had decision making autonomy ^a^	63.9 ± 27.5	-0.1*	1.0	
C. Percentage of women with secondary education or higher ^a^	28.3 ± 24.1	0.0	0.1	1.0

SD, Standard Deviation

According to our bivariate analyses, all types of IPV were significantly associated with overweight. Only sexual IPV, however, was found to be significantly associated with being underweight. Both being underweight and being overweight were significantly associated with decision-making autonomy. All community-level variables were statistically associated with both being underweight and overweight (Table 3).

**Table 3:** Distribution of married women with their nutritional status in Ethiopia

	Characteristics		Underweight		Overweight	
			No %	Yes %	No %	Yes %
**Individual-level characteristics (n = 3,660)**						
	Intimate partner violence (IPV)					
	Physical IPV					
		No	73.10	26.90	92.52	7.48*
		Yes	71.99	28.01	88.75	11.25
	Emotional IPV					
		No	73.11	26.89	92.49	7.51*
		Yes	72.07	27.93	88.66	11.34
	Sexual IPV					
		No	73.26	26.74*	97.20	2.80*
		Yes	68.00	32.00	88.89	11.11
	Any IPV					
		No	73.42	26.58	92.68	7.32*
		Yes	71.58	28.42	88.18	11.82
	Decision-making autonomy					
		No	70.04	29.96*	87.94	12.06*
		Yes	74.58	25.42	92.03	7.97
	Age group of the respondent (years)					
		15–24	67.71	32.29*	95.70	4.30*
		25–34	76.42	23.5	89.28	10.72
		35–49	71.97	28.03	85.24	14.76
	Religion					
		Orthodox	77.98	22.02*	86.16	13.84*
		Muslim	68.73	31.27	89.99	10.01
		Protestant	71.70	28.30	93.90	6.10
		others	78.46	21.54	98.46	1.54
	Place of residence					
		Urban	83.94	16.06*	70.25	29.75*
		Rural	69.38	30.62	95.57	4.43
	Education level of respondent					
		No education	69.49	30.51*	94.14	5.86*
		Primary	76.44	23.56	88.22	11.78
		Secondary or higher	80.40	19.60	71.80	28.20
	Employment status of respondent					
		No	71.24	28.76	91.46	8.54
		Yes	76.10	23.90	85.57	14.43
	Total children ever born					
		0	73.27	26.73*	88.45	11.55*
		1–2	75.70	24.30	86.54	13.46
		3–4	73.09	26.91	89.13	10.87
		≥ 5	70.33	29.67	92.35	7.65
	Contraceptive use					
		No	69.28	30.72*	90.94	9.06*
		Yes	79.56	20.44	86.71	13.29
	Wealth index					
		Poorer	61.01	38.99*	95.50	4.50*
		Poor	72.11	27.89	97.62	2.38
		Medium	73.35	26.65	97.16	2.84
		Rich	77.94	22.06	94.54	5.46
		richer	83.93	16.07	71.14	28.86
**Community-level characteristics (n = 636)**						
	Percentage of women who had experienced any IPV					
		Low	58.62	41.38*	55.64	44.36*
		High	56.2	43.80	72.64	27.36
	Percentage of women who had decision making autonomy					
		Low	42.75	57.25*	46.55	53.45*
		High	49.62	50.38	32.35	67.65
	Percentage of women with secondary education or higher					
		Low	53.93	46.07*	57.30	42.70*
		High	61.60	38.40	47.86	52.14

*P < 0.05

Table 4 summarizes the results of our multilevel analyses for the outcome of underweight. Model 1 (empty model) revealed that 41% of the variation in underweight among non-pregnant married women was attributed to community-level factors (ICC = 0.41; P < 0.005). The analyses of underweight with only individual-level variables (model 2) revealed that sexual IPV, any IPV and decision-making autonomy had a statistically significant association with being underweight. Model 2 also revealed that differences at the community-level accounted for 32% of the variation in underweight (ICC = 0.32; P < 0.05). Model 3 demonstrated that those only community-level variables with the outcome of underweight. The percentage of women in the community who had decision-making autonomy and who completed secondary or higher education were negatively associated with being underweight. In the final model (model 4), we included both individual and community-level variables at the same time to investigate their relationship with the outcome of underweight. Among individual-level factors sexual IPV and decision-making autonomy were found to be significantly associated with being underweight. Women who experienced sexual IPV were 2.97 times more likely to be underweight (AOR = 2.97; 95% CI: 2.02–4.38) than women who had not experienced sexual IPV. Respondents with decision-making autonomy at home had a 17% lower odds of being underweight (AOR = 0.83; 95% CI: 0.69–0.98) than their counterparts. There was no community-level variable found to be significantly associated with being underweight. The ICC in the final model suggests that other unobserved community-level factors may account for 26% of the variation in underweight (ICC = 0.26; P < 0.05).

**Table 4:** Multilevel logistic regression analysesa of factors associated with underweight among married women in Ethiopia

Characteristics			Underweight			
			Model 1	Model 2	Model 3	Model 4
**Individual-level characteristics (n = 3,660)**				AOR(95% CI)^b^	AOR(95% CI)^b^	AOR(95% CI)^b^
	**Intimate partner violence (IPV)**					
	Physical IPV (ref = no)					
		Yes		0.61(0.45–1.01)		0.66(0.45–1.12)
	Emotional IPV (ref = no )					
		Yes		0.82(0.55–1.23)		0.83(0.55–1.23)
	Sexual IPV (ref = no )					
		Yes		2.99(2.03–4.44)*		2.97(2.02–4.38)*
	Any IPV (ref = no)					
		Yes		1.13(1.07–1.82)*		1.13(0.70–1.81)
	Decision-making autonomy (ref = no)					
		Yes		0.82(0.65–0.72)*		0.83(0.69–0.98)*
**Community-level characteristics (n = 636)**						
	Percentage of women who had experienced any IPV (ref = low)					
		High			1.33(0.79–2.35)	1.17(0.70–1.96)
	Percentage of women who had decision making autonomy (ref = low)					
		High			0.55(0.33–0.83)*	0.90(0.53–1.51)
	Percentage of women with secondary education or higher (ref = low)					
		High			0.45(0.27–0.76)*	0.78(0.46–1.33)
**Measure of variation**						
	ICC		0.41	0.32	0.37	0.26
	AIC		9028.91	8843.17	9018.02	8847.79

Note: * indicates the statistical significance values (*p < 0.05.).

^a^ All models are adjusted for, age of respondent, religion, place of residence, education level of respondent, employment of respondent, number of children ever born, wealth index and contraceptive use.

^b^ OR = odds ratio; CI = confidence interval.

^C^ICC= intraclass correlation coefficient, represents the variation in nutritional status among communities

^d^ AIC = Akaike information criterion, a lower AIC value indicates that the model is better fit.

Table 5 depicts the result of multilevel analyses for the outcome of overweight/obesity. In the final model (model 4), we included both individual and community-level variables at the same time to investigate their relationship with overweight/obesity. No individual-level variables were found to be significantly associated with overweight/obesity. Community-level women’s decision-making power was the only variable which was significantly associated with overweight/obesity. Women from communities with a larger proportion of decision-making power women had a 25% reduced likelihood of being overweight/obese (AOR = 0.75; 95% CI: 0.34–0.89) than women from communities with a lower proportion of decision-making power women. According to the ICC in the final model, other unobserved community-level factors may account for 32% of the variation in overweight/obesity (ICC = 0.32; P < 0.05).

**Table 5:** Multilevel analyses of factors associated with overweight/obese among married women in Ethiopia

Characteristics			Overweight			
			Model 1	Model 2	Model 3	Model 4
**Individual-level characteristics (n = 3,660)**				AOR(95%CI)^b^	AOR(95%CI)^b^	AOR(95% CI)^b^
	Intimate partner violence (IPV)					
	Physical IPV (ref = no)					
		Yes		1.57(0.74–3.44)		1.55(0.71–3.40)
	Emotional IPV (ref = no )					
		Yes		0.48(0.21–1.08)		0.49(0.22–1.09)
	Sexual IPV (ref = no )					
		Yes		1.11(0.03–1.34)		1.01(0.03–1.45)
	Any IPV (ref = no)					
		Yes		0.53(0.19–1.45)		0.53(0.19–1.14)
	Decision-making (ref = no)					
		Yes		1.18(0.81–1.72)		1.22(0.83–1.79)
**Community-level characteristics (n = 636)**						
	Percentage of women who had experienced any IPV (ref = low)					
		High			1.40(0.17–1.94)	1.12(0.51–2.41)
	Percentage of women who had decision making autonomy (ref = low)					
		High			0.23(0.13–0.83)*	0.75(0.34–0.89)*
	Percentage of women with secondary education or higher (ref = low)					
		High			2.06(0.87–4.98)	0.69(0.31–1.52)
**Measure of variation**						
	ICC		0.64	0.38	0.44	0.32
	AIC		4185.02	4175.05	4168.49	3496.89

Note: * indicates the statistical significance values (*p < 0.05.).

^a^ All models are adjusted for, age of respondent, religion, place of residence, education level of respondent, employment of respondent, number of children ever born, wealth index and contraceptive use.

^b^ OR = odds ratio; CI = confidence interval.

^C^ICC= intraclass correlation coefficient, represents the variation in nutritional status among communities

^d^ AIC = Akaike information criterion, a lower AIC value indicates that the model is better fit.

## Discussion

This study establishes evidence for an association between IPV and decision-making autonomy on women’s nutritional status in Ethiopia, based on nationally representative data. About 28.4% of women had ever experienced physical, emotional or sexual or all forms of violence from their husband/partner. According to a 2021 WHO report, the prevalence of IPV in our study was slightly lower than the global prevalence, which was 30% of women worldwide. The issue of IPV especially sexual IPV is sensitive in Ethiopian society, particularly in rural areas, this may be connected with negative sentiments of shame and stigma, and because a major proportion of our study sample was from rural areas, there is a possibility of underreporting. Around 32% of women had no decision-making autonomy at home.

Among the sampled women malnutrition is a serious problem: 27.0% of women were suffering from being underweight and 10.6% of participants were overweight/obesity. These findings suggest that both underweight and overweight/obese coexist among Ethiopian women. Malnutrition (underweight and overweight) is more prevalent in our study participants than in the national survey. According to the 2016 EDHS, 22% of women aged 15–49 are underweight, while 8% are overweight or obese [21]. The possible explanation for this discrepancy is that our study only included non-pregnant married women of reproductive age. However, the 2016 EDHS included all women of reproductive age.

After adjusting socio-demographic and economic factors, our final model discovered that women who had experienced sexual IPV were more likely to be under-nutrition than their counterparts. IPV against women can cause both immediate and long-term psychosomatic symptoms, which may disturb their dietary consumption behaviors. Loss of appetite, eating disorders, and physical inactivity, are among them [30]. IPV can also increase the risk of women having poor nutritional status by controlling food from abusive husbands. In addition, IPV is strongly linked to a woman’s inability to make decisions for herself and her family; including the qualities and quantities of food she prepares [31]. According to Elaine M. Power’s research, women who have economically abusive intimate partner are more likely to live in poverty and face food insecurity. Economic abuse generated food insecurity, which coexisted with physical, psychological, and sexual abuse. Furthermore, women in abusive marriages employ a number of techniques, including going hungry themselves, to save their children from going hungry [32]. As a result of this condition, women suffer from under-nutrition.

The study conducted in Bangladesh and India also shows that women who have experienced IPV have a higher risk of malnutrition [33, 34]. The observed association between IPV and under-nutrition may involve a mediating effect of psychological stress [30]. Stress plays a significant role in contributing to being underweight by influencing women’s eating habits, such as appetite loss, irregular meal, and eating too little, all of which increase women’s risk of under-nutrition [35]. The alternative explanation for the association between IPV and malnutrition may be withholding of food as a form of abuse. This suggests that preventing intimate partner violence is an important component not only from an amoral and intrinsic viewpoint but also because of the instrumental health benefits [36].

Our study found that individual-level women’s decision-making autonomy was inversely related to under-nutrition. Indicating that gender equality, as measured by women’s decision-making autonomy, can have an impact on women’s nutritional status. Women’s participation in household decision-making provides them with the opportunity to purchase food, which improves the nutritional status of women, their children, and the entire family [37, 38]. This is because women with more financial independence have more bargaining power when it comes to food purchases [39]. Women’s ability to feed themselves and their family had been severely hampered by a lack of financial resources as a result of economic abuse [32].

Evidences suggest that women’s decision-making autonomy improves child nutrition as well [40–42]. Autonomous Women can play a crucial role in the alteration of intra-household dynamics that can result in improved health and nutrition outcomes especially for children. This shows that ensuring women’s decision-making power at home should be a significant component in improving their and their families’ nutritional status [43]. Empowering women and improving nutritional status of the population are two essential components of the Sustainable Development Goals (SDGs 2 and 5) targeted for achievement by 2030 [44, 45]. Our study suggests that efforts to achieve Sustainable Development Goal (SDG 5) have an impact on other SDGs particularly SDG2 [14].

The effect of community-level decision-making power was found to have a significant association with overweight/obesity among community-level variables. After controlling for their decision-making status and other individual and community-level factors, women living in a community with a high proportion of women with high decision-making status were less likely to be overweight/obese. This could be because social relationships in Ethiopia are close, and thus the effect of community-level autonomy on individual behavior may overshadow individual autonomy. Despite the fact that most Ethiopian women have limited decision-making power, living in a community with empowered women can lead to increased use of shared information and resources to improve nutritional status [15]. The presence of a large number of autonomous women in a community may help to disseminate information about better health outcomes to those with lower levels of autonomy via formal and informal social networks [25]. The literature also shows that peer-communication in the community on health behaviour has positive outcomes [46, 47]. This implies that female decision-making power at the community-level had a beneficial effect on nutrition improvement.

There may be some limitations to this study. First, due to the cross sectional natures of the study we cannot confirm the causality between IPV and decision making power on nutritional status. Second, the potential for underreporting is a crucial concern in research on IPV because of the sensitivity, social stigma, and safety concerns regarding this topic. Third, we use only BMI to measure nutritional status, which depends upon weight and the square of height cannot differentiate between lean and fat mass and cannot identify fat distribution, which is a significant component in metabolic health risk [48, 49]. This suggests that the future study should use BMI in combination with waist circumference to increase validity and accurate reporting of nutritional status. Despite the above limitations, our study used a national representative large sample of non-pregnant women at reproductive age, and therefore, results from the current analysis generalized to Ethiopian non-pregnant women at reproductive age.

## Conclusion

Our study demonstrates the importance of considering both individual-and community-level characteristics when investigating the effects of IPV and decision making power on women’s nutritional status. This study revealed the presence of a significant association between IPV and decision making power with women’s nutritional status. These findings suggest that efforts to achieve Sustainable Development Goal (SDG) 5 (gender equality) could have an impact on achieving other SDGs including SDG1-4. As a result, effective policies and programs are required to end the practice of violence against women and girls and encourage them to participate in decision-making in order to improve women’s nutritional status and consequently achieve SDGs.

## Data Availability

The datasets used in this study can be found at: https://dhsprogram.com/data/available-datasets.cfm.

## Abbreviations

AIC: Akaike information criterion
AOR: Adjusted Odds ratio
BMI: Body Mass Index
CI: Confidence Interval
DHS: Demographic and Health Survey
ICC: Intraclass correlation coefficient
IPV: Intimate Partner Violence
LMIC: Low and Middle Income Countries
SDG: Sustainable Development Goal
